# The RNA cap methyltransferases RNMT and CMTR1 co-ordinate gene expression during neural differentiation

**DOI:** 10.1042/BST20221154

**Published:** 2023-05-05

**Authors:** Shang Liang, Rajaei Almohammed, Victoria H. Cowling

**Affiliations:** 1 Cancer Research UK Beatson Institute, Glasgow, U.K.; 2 School of Cancer Sciences, University of Glasgow, Glasgow G61 1BD, U.K.

**Keywords:** CMTR1, differentiation, embryonic stem cells, gene expression, RNA cap, RNMT

## Abstract

Regulation of RNA cap formation has potent impacts on gene regulation, controlling which transcripts are expressed, processed and translated into protein. Recently, the RNA cap methyltransferases RNA guanine-7 methyltransferase (RNMT) and cap-specific mRNA (nucleoside-2'-O-)-methyltransferase 1 (CMTR1) have been found to be independently regulated during embryonic stem (ES) cell differentiation controlling the expression of overlapping and distinct protein families. During neural differentiation, RNMT is repressed and CMTR1 is up-regulated. RNMT promotes expression of the pluripotency-associated gene products; repression of the RNMT complex (RNMT–RAM) is required for repression of these RNAs and proteins during differentiation. The predominant RNA targets of CMTR1 encode the histones and ribosomal proteins (RPs). CMTR1 up-regulation is required to maintain the expression of histones and RPs during differentiation and to maintain DNA replication, RNA translation and cell proliferation. Thus the co-ordinate regulation of RNMT and CMTR1 is required for different aspects of ES cell differentiation. In this review, we discuss the mechanisms by which RNMT and CMTR1 are independently regulated during ES cell differentiation and explore how this influences the co-ordinated gene regulation required of emerging cell lineages.

## Introduction

**Figure 1.**
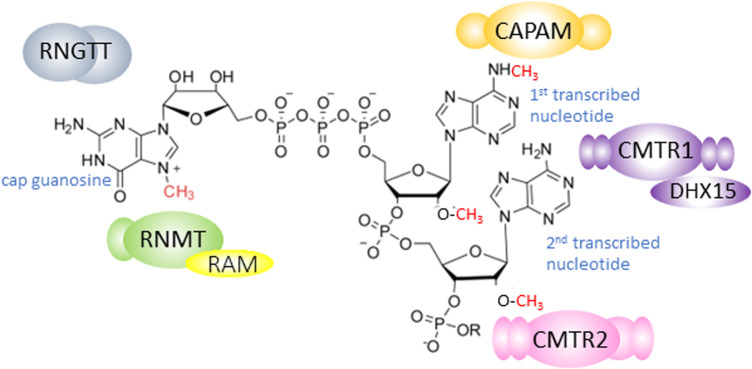
The mammalian RNA cap. Diagram of a RNA cap structure in mammals. An inverted 7-methyl guanosine is linked to the first transcribed nucleotide via a triphosphate bridge. The first and second transcribed nucleotides are methylated on the O-2 position of the ribose. When the first transcribed nucleotide is adenosine, it can be methylated on the N-6 position as depicted here. The capping enzyme RNGTT adds the inverted guanosine to the first transcribed nucleotide. RNMT methylates the cap guanosine on the N-7 position. CMTR1 and CMTR2 methylate the ribose on the O-2 position of the first and second transcribed nucleotides, respectively. When the first transcribed nucleotide is adenosine, CAPAM methylates the N-6 position. RNMT is depicted interacting with its activating cofactor RAM. CMTR1 is depicted interacting with its activity modulator DHX15.

**Figure 2.**
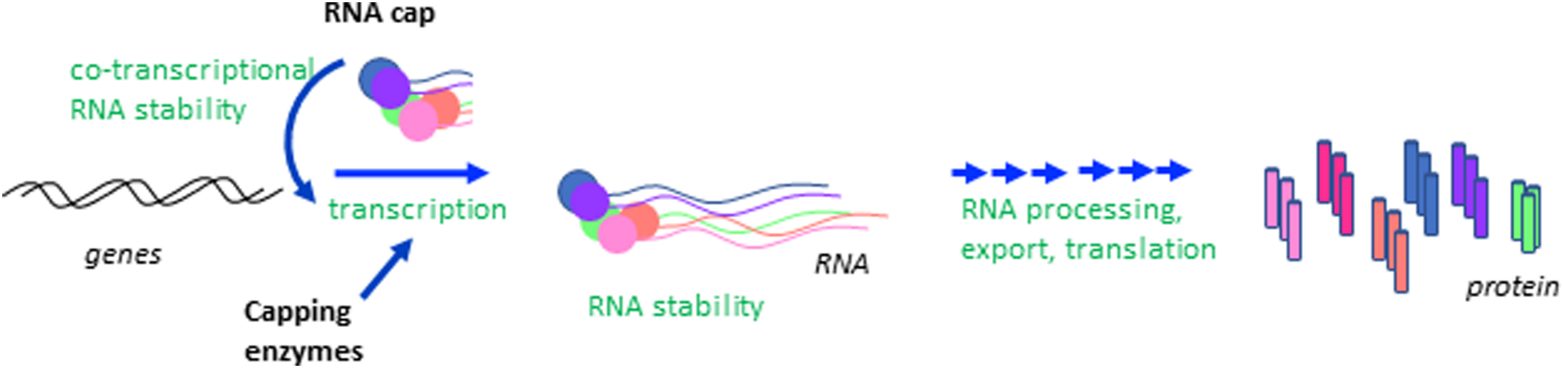
The RNA cap impacts on multiple steps in gene expression. The RNA cap is formed co-transcriptionally on the 5′ end of RNA polymerase II transcripts. The RNA cap protects nascent transcripts from degradation during synthesis and the RNA capping enzymes have roles in promoting transcription. The RNA cap protects mature mRNA from degradation in the cytoplasm, including by preventing recognition by factors of the innate immune response. The RNA cap recruits factors involved in RNA processing, nuclear export and translation initiation.

Uncovering the molecular mechanisms operating in embryonic stem (ES) cells is critical for our understanding of development and many disease states, and for the use of these cells in therapeutics. The function and fate of ES cells are governed by networks of transcription and translation which are co-ordinately controlled during differentiation programmes [1,2]. As in all mammalian cells, in ES cells the synthesis, processing and translation of RNA polymerase II (RNA pol II) transcripts are dependent on the RNA cap, a methylated nucleotide structure on the 5′ end (Figure 1) [3,4]. The RNA cap is synthesised on the first transcribed nucleotides, protecting RNA from nucleases and recruiting protein complexes involved in RNA processing and translation initiation [4–7] (Figure 2). Formation of the RNA cap has several mechanistic links to transcription: the cap protects RNA from degradation during transcription and some capping enzymes can promote transcription in mechanisms independent of their catalytic function [8–13]. In recent years, studies in diverse systems have revealed that different genes have distinct dependencies on the RNA cap for their transcripts to be stably expressed, processed and translated [3,7,14–19]. This gene specificity can be biologically potent when regulation of RNA cap formation results in the co-expression of functionally related RNAs and proteins, directing changes in cellular processes and function [8,14,16,20–22]. In this review, we discuss the finding that during ES cell differentiation, repression of the cap methyltransferase complex RNMT–RAM and up-regulation of the cap methyltransferase CMTR1 are required to co-ordinate the expression of RNAs and proteins associated with pluripotency, cell growth and cell proliferation [23,24].

## Formation of the RNA cap by the RNA capping enzymes

The mammalian RNA cap is formed by a series of enzymes [3,4,25] (Figure 1). During the early stages of transcription, the sequential action of a triphosphatase and guanylyltransferase in the enzyme RNGTT (RNA guanylytransferase and triphosphatase) joins the inverted guanosine cap to the first transcribed nucleotide, via a triphosphate bridge (Figure 2). RNGTT is positioned on the RNA pol II complex to act on nascent RNA as it emerges [26–28]. Guanosine cap addition is important for RNA protection during transcription, permitting full-length transcripts to be synthesised (Figure 2) [29,30]. Subsequently, a series of cap methyltransferases, RNMT (RNA guanine-7 methyltransferase), CMTR1 (cap-specific mRNA (nucleoside-2′-O-)-methyltransferase 1), CMTR2 (cap-specific mRNA (nucleoside-2′-O-)-methyltransferase 2) and CAPAM (cap-specific adenosine methyltransferase) methylate specific sites on the guanosine cap and first two transcribed nucleotides [4,25,31] (Figure 1). RAM is the activating subunit of the RNMT complex [32]. N-7 cap methylation prevents the removal of the guanosine cap during transcription, thus contributing to the co-transcriptional stabilisation of nascent RNA [29,33]. N-7 cap methylation and the other cap methylations also provide the cap with a structure uniquely found on RNA pol II-transcribed RNA which can thus specifically recruit processing factors to these transcripts [4,34]. The methylated inverted guanosine cap and initial two transcribed nucleotides are currently recognised to constitute the RNA cap in mammals (Figure 1) [35]. This definition may be expanded as new RNA modifications are discovered.

**Figure 3.**
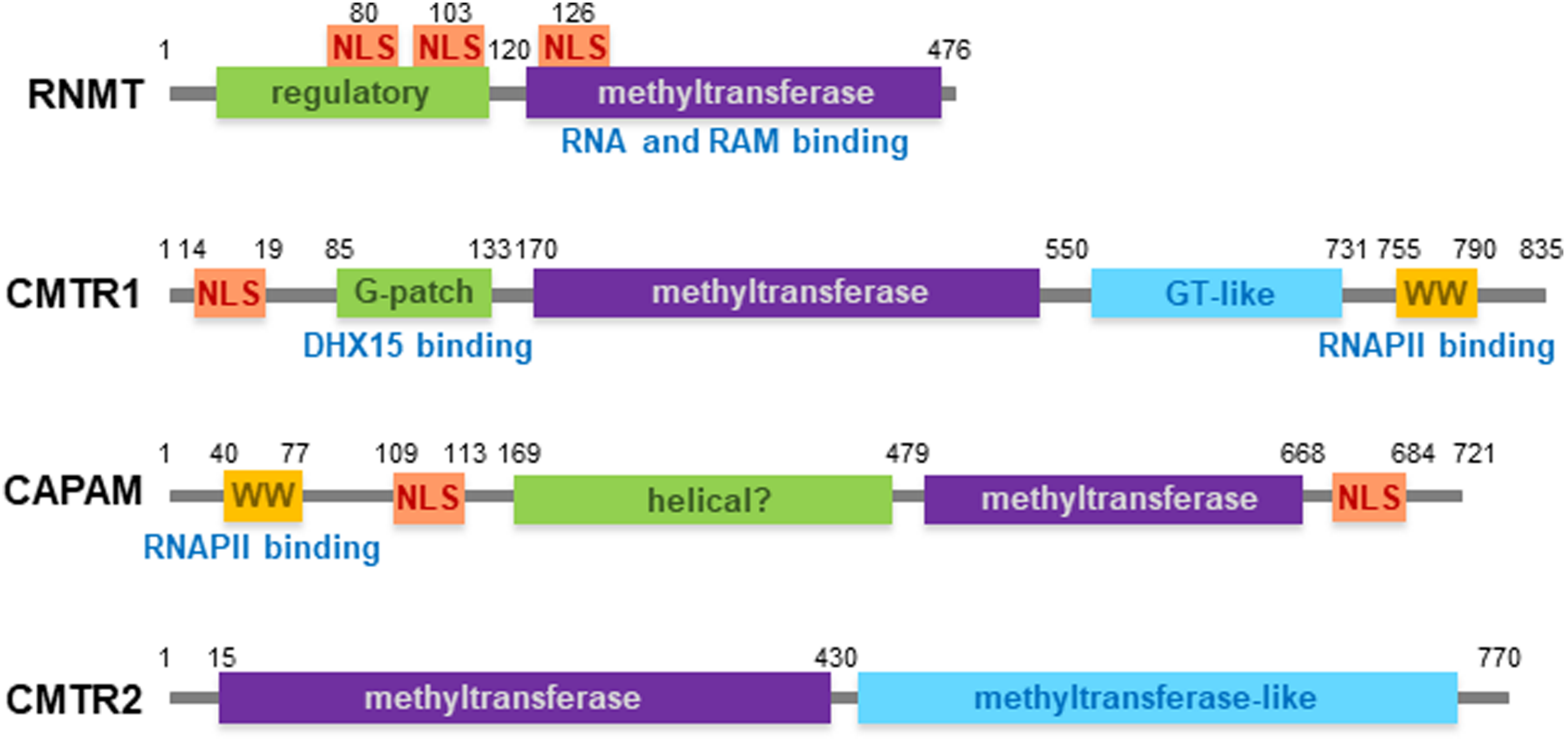
The functional domains of the RNA cap methyltransferases. Amino acid positions and functional domains indicated. Domains of interaction indicated; RNA polymerase II (RNAPII) binds CMTR1 and CAPAM WW domains, RNA binds RNMT methyltransferase domain/RAM, RAM binds RNMT methyltransferase domain and DHX15 binds CMTR1 G-patch.

Methylation of the cap nucleotides by RNMT, CMTR1 and CAPAM occurs predominantly co-transcriptionally when the enzymes are recruited to the nascent cap, RNA and/or RNA pol II [36–39]. CMTR2 methylates the RNA cap predominantly in the cytoplasm and other capping enzymes have also been observed to function post-transcriptionally [7,40–42]. The RNA cap methyltransferases are expressed as distinct enzymes in mammals and therefore can function and be regulated independently (Figure 1) [3,25,31]. The enzymes have similar methyltransferase domains, but these are flanked by functional domains which are different in each cap methyltransferase (Figure 3). The distinct configurations of the RNA cap methyltransferases facilitate their independent mechanisms of action and independent regulation by cofactors and post-translational modifications [18,19,37,40,43–45]. As discussed, some of the capping enzymes have been demonstrated to have catalysis-independent functions, most obviously in transcription although they may have roles in other RNA processing events as well (Figure 2) [7–13].

## RNMT and CMTR1 regulate different target RNAs in embryonic stem cells

In ES cells, the pluripotent state is maintained by open chromatin and transcriptional plasticity [46]. During differentiation, regulation of chromatin accessibility and transcription specifies emergent cell identities. The role of the two major RNA cap methyltransferases, RNMT and CMTR1, was investigated during ES cell differentiation [23,24]. RNMT and CMTR1 can be described as the major cap methyltransferases because the majority of mature mRNA (in ESCs and other mammalian cells) carries both methylations catalysed by these enzymes [47,48]. RNMT catalyses methylation of the guanosine cap at the N-7 position, a modification important for binding to the cap-binding complexes, including CBC and eIF4F, and for protecting RNA from degradation, including during transcription [4,6,21,34]. RNMT also has methylation-independent roles in promoting transcription, via the recruitment of transcriptional regulators to the RNA pol II complex [8]. Other N-7 guanosine cap methyltransferases also have non-catalytic roles in transcription although the mechanisms involved vary [9,10,49]. CMTR1 catalyses O-2 methylation of the first transcribed nucleotide ribose, a modification which was initially linked to RNA translation, although recent genetic studies have demonstrated more predominant roles in RNA synthesis and stability [14–16,18,24,50–52]. CMTR1 forms a complex with the RNA helicase DHX15, with both enzymes influencing the action of each other [39,52]. How ribose O-2 methylation influences RNA cap-binding protein recruitment and impacts on RNA processing events is less well described. Recent discoveries reveal that, as with N-7 cap guanosine methylation, the impacts of cap ribose O-2 methylation are gene-specific [14–16,18,51].

**Figure 4.**
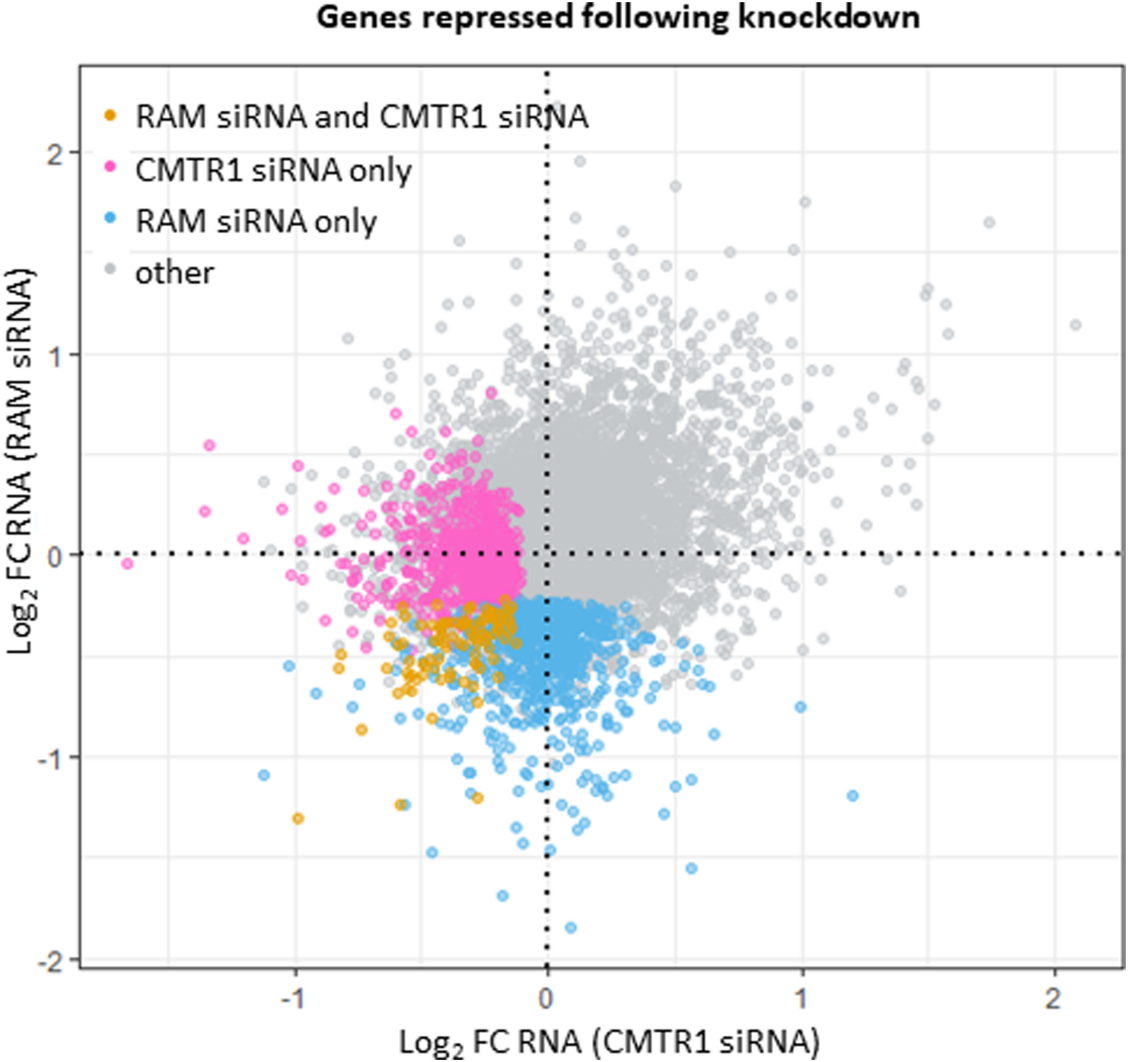
RNA expression analysis following siRNA targeting of RAM or CMTR1. RNMT–RAM and CMTR1 were repressed in ES cells following siRNA transfection targeting RAM or CMTR1. RNA was analysed by RNA sequencing analysis. GEO RNA-seq data series GSE81849 with the samples of control (GSM2176662–2176665) and RAM siRNA-treated cells (GSM2176666–2176669) and data series GSE175631 with the samples of control (GSM5432986–5432988) and CMTR1 siRNA-treated cells (GSM5432993–5432996) were used in the analysis. Reads were mapped using STAR and were processed as in [24]. The reads aligned to transcripts were counted with featureCounts. EdgeR was used to analyse the differential RNA expression between RAM siRNA and CMTR1 siRNA transfected cells and their controls. Axes represent log_2_ fold change in RNA relative to control. Genes repressed by transfection of RAM siRNA alone, CMTR1 siRNA alone or both are highlighted (log_2_FC < 0, FDR < 0.05).

In ES cells, the transcripts whose expression is most dependent on RNMT and CMTR1 were analysed by RNA sequencing [23,24]. It is useful to compare these datasets. The RNMT–RAM complex was repressed using RAM siRNA, mimicking the mechanism of RNMT–RAM suppression in ES cell differentiation [23]. CMTR1 expression was repressed directly by siRNA [24]. These datasets were produced at different times using similar cell culture and molecular biology protocols. Both datasets were prepared following the transfection of a single siRNA, and the regulation of a selection of genes was validated using other independent siRNAs and gene editing [23,24]. Some variation in the control sets of genes was observed, but overall the control transfection datasets correlate well (data not shown). In the following description of the analysis, the term gene transcripts is used to describe the products of each single gene. Of the 13 213 gene transcripts with expression above background in both experiments, 802 were only repressed in response to RAM siRNA whereas 718 were only repressed in response to CMTR1 siRNA (Figure 4, Supplemental Table S1). Ninety-six gene transcripts were repressed in both. Note that repression of RNA can be a result of reduced transcription or reduced RNA stability and the RNA cap and capping enzymes can have direct and indirect roles in both, as detailed above. Therefore, in ES cells RNMT and CMTR1 regulate fewer common RNAs than distinct RNAs.

## RNMT and CMTR1 are recruited to the immature RNA cap by distinct mechanisms

**Figure 5.**
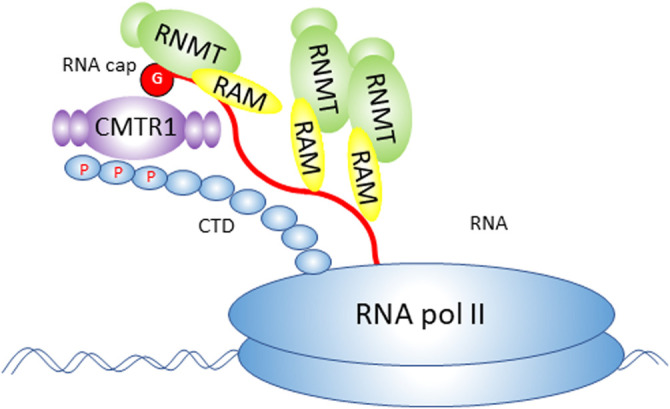
Recruitment of RNMT–RAM and CMTR1 to the immature guanosine cap. The active sites of RNMT and CMTR1 bind to the immature guanosine cap. RNMT recruitment to the cap is enhanced by the RNA binding/activating subunit RAM. RNMT–RAM also binds the full length of the nascent transcript via RAM. CMTR1 recruitment to the cap is by the WW domain binding directly to RNA polymerase II C-terminal domain (CTD).

How do RNMT–RAM and CMTR1 regulate different RNAs [23,24]? Although RNMT and CMTR1 methylate different positions on the same RNA cap substrate, their mechanisms of action are distinct. We may gain some insight into how RNMT and CMTR1 influence the expression of different genes and RNAs by looking at how these enzymes are recruited to the cap (Figure 5). RNMT–RNA interactions were mapped in HeLa cells using CLIP (UV cross-linking and immunoprecipitation), a technique in which RNA–protein complexes are isolated and the RNA is sequenced [12]. This revealed that RNMT binds predominantly to RNA during transcription binding along the full length of the transcript [12]. Unlike most other RNA capping enzymes, direct interaction of RNMT with the RNA pol II complex has not been detected in cell extracts or with recombinant proteins, despite extensive efforts [8,43]. RNMT does not have a WW domain, through which CAPAM and CMTR1 interact with the RNA pol II C-terminal domain (CTD) directly [37,39]. In ChIP (chromatin immunoprecipitation assays), minimal interaction of RNMT with the transcription start site is observed, and this is likely to involve direct interactions with the guanosine cap and RNA, rather than direct interactions with RNA pol II [24,36]. RNMT influences transcript abundance in a gene-specific manner, including via impacts on RNA stability and via methylation-independent impacts on transcription [3,8,21]. CMTR1 interacts directly with the RNA pol II CTD via the WW domain, binding with preference to S5 phosphorylated CTD [38,39] (Figure 4). CMTR1 is recruited effectively to the transcription start site in correlation with RNA pol II abundance [24]. CMTR1 also has gene-specific impact on transcript abundance correlating with RNA pol II occupancy on the TSS [24]. RNMT and CMTR1 have potent impacts on ES cell function and therefore are also likely to have indirect impacts on the expression of genes [23,24].

Since RNMT binds to nascent RNA as it is being transcribed by RNA pol II [8], and CMTR1 binds directly to Ser-5 phosphorylated CTD [39], it is perhaps unsurprising that there is significant over-lap in transcripts dependent on these enzymes for expression level. However, due to the dynamics of RNA synthesis and degradation, and due to the dynamics of CTD S5 phosphorylation, there can be discordance between the abundance of S5 phosphorylated CTD at the TSS of a specific gene and the transcript abundance of that gene. This may, in part, account for the difference in which transcripts are most dependent on RNMT and CMTR1 for expression, processing and stability [23,24].

Other determinants of the gene expression response to RNMT and CMTR1, include their methylation-independent functions. This has been documented for RNMT most clearly [8], and previously in the *Saccharomyces cerevisiae* homologue of RNMT, *ABD1* [9], and the *Schizosaccharomyces pombe* homologue of RNMT, *PCM1* [10,49]. RNMT can promote transcription in a catalytic-independent manner, both in cells and *in vitro* in nuclear run-on assays [8]. In these assays, ribosomes are not present and therefore nascent transcription is analysed independently of nascent protein production. RNMT binds to a series of complexes involved in transcription, including the PAF complex whose recruitment to chromatin it promotes, likely via protein : protein interactions [8]. Other protein complexes may be involved in RNMT-dependent transcription and this may vary in a cell and gene-specific manner, dependent on the expression of the complexes involved and their role in gene transcription [9,10,49]. CMTR1 can also promote transcription elongation in isolated nuclei in run-on assays, although the mechanism has not been explored [24]. Of note, the drosophila homologue of another cap methyltransferase, CAPAM, is not catalytically active and the enzyme was originally identified in mammalian systems as a RNA pol II binding transcriptional regulator [53–55].

## Repression of RNMT and up-regulation of CMTR1 are crucial for embryonic stem cell differentiation

**Figure 6.**
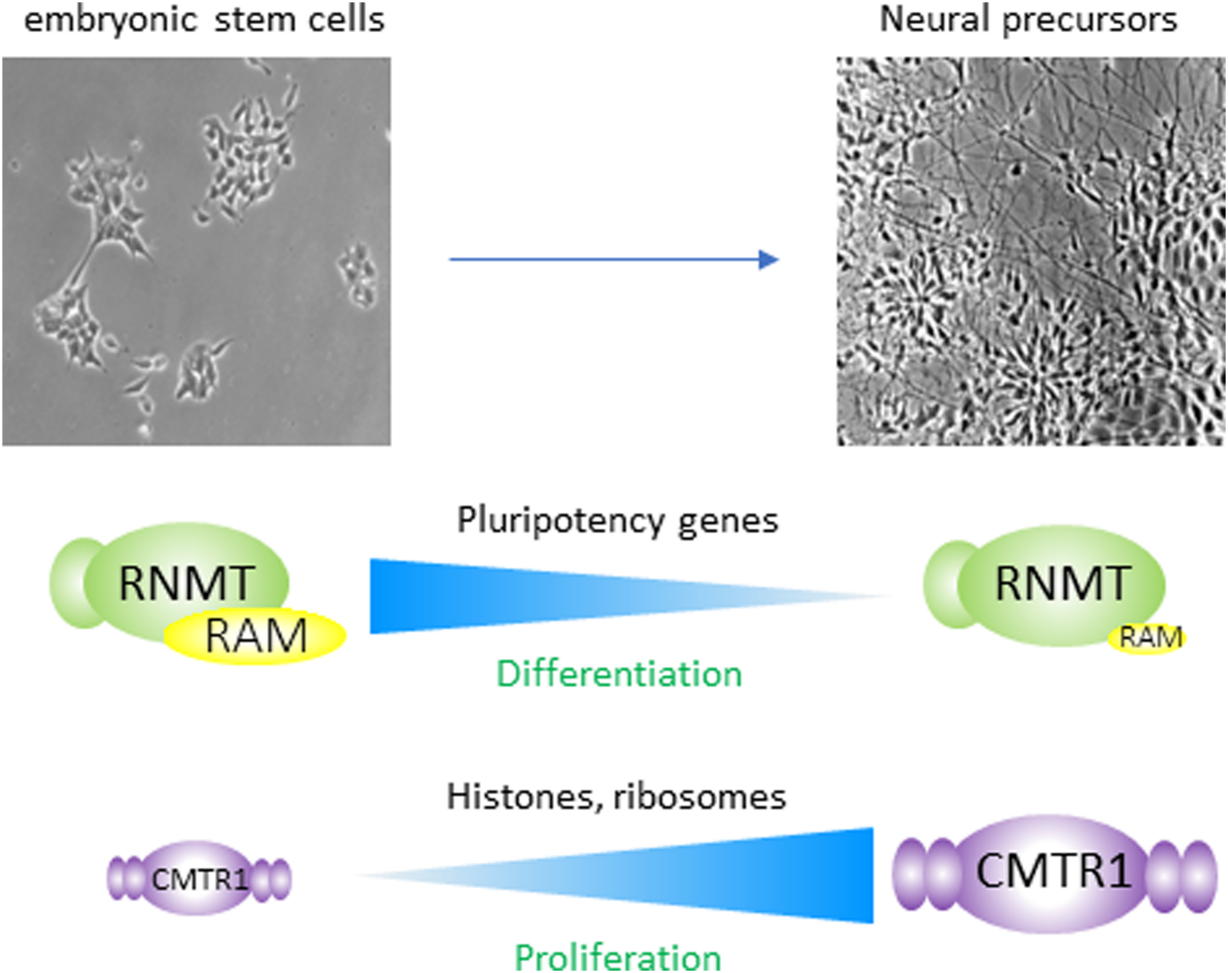
Regulation of RNMT and CMTR1 during embryonic stem cells differentiation. When mouse embryonic stem cells are induced to differentiate to a neural fate RNMT–RAM is repressed; ERK1/2 phosphorylation of RAM induces its ubiquitination and degradation. RNMT–RAM enhances the expression of pluripotency-associated genes. During differentiation, repression of RNMT–RAM is required for repression of pluripotency-associated genes and for differentiation to occur. CMTR1 is up-regulated during differentiation. The predominant targets of CMTR1 are the histone and ribosomal protein genes. During differentiation, CMTR1 is required to maintain expression of these genes and indirectly to maintain translation, DNA replication and proliferation.

ES cell differentiation is dependent on the co-ordinated regulation of specific subsets of genes associated with pluripotency and emergent cell lineages. RNMT and CMTR1 were found to be distinctly regulated during the neural differentiation of ESCs and to have potent impacts on gene expression during this process [23,24] (Figure 6). RNMT and its activating cofactor RAM are highly expressed in ES cells [23]. RAM binds to the catalytic domain of RNMT, increasing catalytic activity, increasing the interaction of the RNMT complex with RNA and increasing RNMT stability [32,56-58]. During ES cell differentiation, the kinases ERK1 and ERK2 are up-regulated and phosphorylation of their targets co-ordinates molecular events during the process [59–61]. RAM is phosphorylated by ERK1/2 during the first few days of differentiation, targeting it for ubiquitination and proteosome-mediated degradation [23]. RNMT action is, therefore, repressed during differentiation resulting in reduced RNA binding and cap methyltransferase activity. Conversely, CMTR1 has relatively low expression in ESCs and is up-regulated gradually during differentiation [24]. The pathways controlling CMTR1 are less well defined, but it is up-regulated by a post-transcriptional mechanism.

One of the key families responsive to RNMT–RAM in ES cells is the pluripotency-associated proteins (Figure 6) [23]. Experimental maintenance of RAM during differentiation prevented certain pluripotency factors including Oct4 and Sox2 from being repressed and differentiation was inhibited. How does RNMT–RAM control specific sets of proteins, including those associated with pluripotency? RNMT–RAM binding to RNA has been mapped in CLIP assays in HeLa cells [12]. In HeLa cells, RNMT–RAM binds to RNA pol II transcripts with little gene specificity, with correlation to abundance. In these cells, RNMT was found to influence the expression level of RNAs in correlation with their abundance, with a small subset of exceptions [8] (Figure 5). In ES cells, RNMT–RAM may control the expression of pluripotency-associated genes, in part because as a gene family, they are relatively high in RNA abundance. Other factors which may influence RNMT–RAM gene-specific impact on RNA levels is the abundance of cap-binding proteins which directly control RNA stability or indirectly control RNA stability by enhancing RNA processing and translocation [4,6]. Of note in T cells, a subset of RNMT-responsive transcripts binds to LARP1, a cap-binding protein which stabilises RNAs with a m7G cap and polypyrimidine tract in the 5′ untranslated region (UTR), described as TOP RNAs [21]. Although the TOP-RNAs are not overtly RNMT-responsive in ESCs, other cap-binding proteins may be mediating the selective repression of transcript levels and processing, including pluripotency-associated transcripts [23]. In addition, some pluripotency-associated proteins may be indirectly responsive to RNMT–RAM suppression during differentiation.

Histone genes and ribosomal protein (RP) genes transcripts are the gene families most dependent on CMTR1 in ES cells [24]. However, repression of CMTR1 by siRNA or CRISPR-mediated depletion only results in mild repression of these RNAs in ES cells and proliferation is largely unaffected [24]. In contrast, during differentiation, the dependency of histone gene and RP gene transcripts on CMTR1 increases. When CMTR1 is experimentally repressed during differentiation, the histone genes and RP genes transcripts are repressed more than in ESCs. In correlation with histone and RP repression, DNA replication checkpoints are engaged, DNA replication stress is observed, translation is reduced and the differentiating cells fail to proliferate [24]. The few ESCs that do survive differentiation when CMTR1 is repressed exhibit normal regulation of pluripotency and differentiation markers, indicating that they are still undergoing a version of differentiation. Why ES cells become more dependent on CMTR1 as they proliferate is unresolved. Chromatin becomes more compact during differentiation, which may result in RNA polymerase II and associated enzymes, including CMTR1, having decreased access to promoters and transcription initiation sites [1,62]. In addition, during differentiation increased RPs are required for translation, which may place increased dependence on CMTR1 up-regulation for the maintenance of RP production [24,63].

## RNMT and CMTR1 regulation of specific RNAs: pluripotency, histones and ribosomal proteins

**Figure 7.**
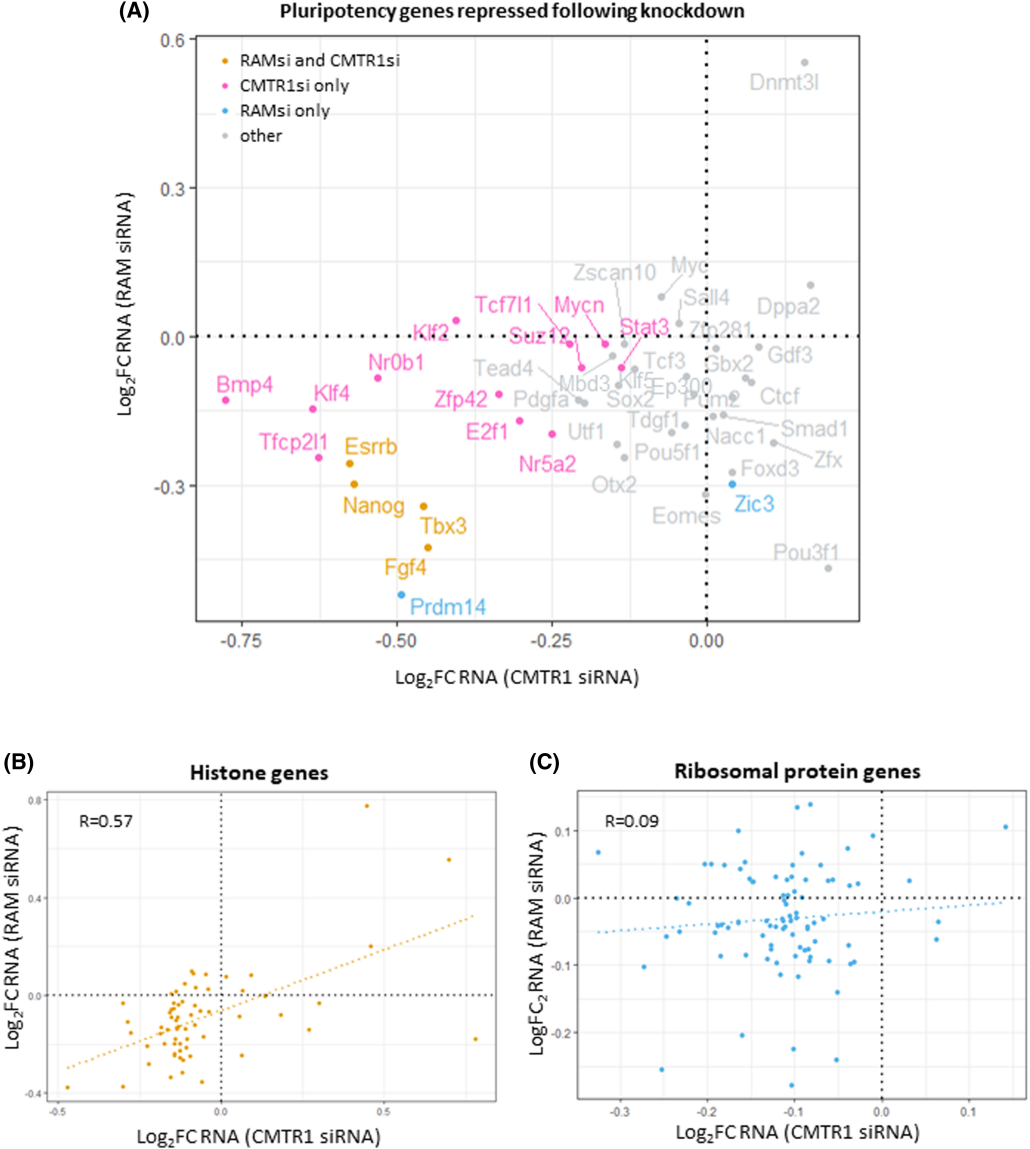
RNA expression analysis following siRNA targeting of RAM or CMTR1. RNMT–RAM and CMTR1 were repressed in ESCs following siRNA treatment targeting RAM or CMTR1. RNA was analysed by RNA sequencing analysis. Axes represent log_2_ fold change in RNA relative to control. (**A**) Repressed pluripotency-associated genes. Genes repressed by transfection of RAM siRNA alone, CMTR1 siRNA alone or both are indicated. (**B**) Histone genes. (**C**) Ribosomal protein genes. A linear regression line and the correlation coefficient (R) were indicated in (**B**) and (**C**).

As discussed, the repression of RNMT–RAM results in the repression of many pluripotency-associated gene transcripts and proteins*,* which can be observed in the metadata analysis and in the analysis of individual RNAs and proteins in ESCs, including during differentiation (Figure 7A) [23]. A subset of pluripotency-associated genes transcripts is also CMTR1 dependent although their protein production is not repressed on CMTR1 CRISPR-mediated depletion, indicating that ribose O-2 methylation is less important for their RNA processing and translation (Figure 7A) [24].

The histones are the predominant CMTR1 target gene transcript family (Figure 7B). Here, we note that the histone gene transcripts are also RNMT–RAM dependent in ES cells and as RNMT–RAM is repressed during differentiation, the histones are repressed unless CMTR1 is up-regulated to maintain their expression [23,24]. As discussed, the RP gene transcripts are also CMTR1 dependent in ES cells (Figure 7C) [24], whereas, as a group these transcripts are not overtly RNMT-dependent in ES cells [23]. This is in contrast to T cells, in which the RP gene transcripts are the most RNMT-dependent gene family [21], and RPs are also RNMT–RAM dependent in HeLa cells [8]. This highlights another interesting facet of RNA cap function and specificity. In different cell lineages, the same cap methyltransferase can have distinct dependent gene transcripts and proteins. Why are the RP transcripts significantly dependent on RNMT–RAM in T cells and HeLa cells but not in ES cells? The RP transcripts are highly abundant in ES cells, indicating that this is not a dominant factor dictating RNMT–RAM dependency in these cells [23,24]. There are many potential explanations; including methylation-independent functions of RNMT–RAM [8], and the relative abundance of cap-binding proteins which mediate the stability or degradation of RNA in ES cells [4,6]. The model emerges that the specific impact of each capping enzyme is dependent on which RNAs are expressed, the configuration of the capping enzymes and their interaction with substrates, and the relative abundance of RNA and cap-binding proteins and RNA degradation enzymes.

## Summary

To conclude, the cap methyltransferases RNMT and CMTR1 control the expression of distinct sets of gene products. By examining their mechanisms of action and how they are recruited to their substrates we can begin to understand why their target genes differ. During ES cell differentiation, the independent regulation of RNMT and CMTR1 facilitates the co-ordinate regulation of the pluripotency-associated genes, histones and RPs required for differentiation and proliferation during this process.

Major cellular events are accompanied by regulation of the RNA capping enzymes, which can direct co-ordinated and coherent regulation of gene expression and influence cell proliferation, cell differentiation and immune responses. Experimentally we can direct capping enzyme functionality and therefore development of therapeutic strategies to modify capping enzyme expression or catalytic activity is possible. Such cap therapeutics may have uses in regenerative medicine, cancer therapeutics and the treatment of neurological disorders, when directed interference in gene regulation has value.

## Perspectives

- Regulation of the RNA cap methyltransferases impacts on many steps in gene expression and indirect impacts on cell functions and cell fate decisions.
- Here, we discuss how RNA cap methyltransferase regulation during ES cell differentiation impacts on RNA expression associated with pluripotency, translation, DNA replication and proliferation.
- Gene expression in other stem cells is likely to be influenced by the regulation of RNA cap methylation, with the specific genes regulated being tissue dependent.

## Open Access

Open access for this article was enabled by the participation of University of Glasgow in an all-inclusive *Read & Publish* agreement with Portland Press and the Biochemical Society under a transformative agreement with JISC.

## Abbreviations

CAPAM: cap-specific adenosine methyltransferase
CMTR1: cap-specific mRNA (nucleoside-2′-O-)-methyltransferase 1
CMTR2: cap-specific mRNA (nucleoside-2′-O-)-methyltransferase 2
CTD: C-terminal domain
ES: embryonic stem
RNA pol II: RNA polymerase II
RNGTT: RNA guanylytransferase and triphosphatase
RP: ribosomal protein
UTR: untranslated region
